# Performance of composite mineral adsorbents for removing Cu, Cd, and Pb ions from polluted water

**DOI:** 10.1038/s41598-019-49857-9

**Published:** 2019-09-19

**Authors:** Woo-Ri Lim, Sung Wook Kim, Chang-Han Lee, Eun-Kyeong Choi, Myoung Hak Oh, Seung Nam Seo, Heung-Jai Park, Se-Yeong Hamm

**Affiliations:** 1 Department of Geological Sciences, Pusan National University, 63beon-gil 2, Geumjeong-gu, Busan, 46241 Republic of Korea; 2 GI Co., Ltd., 11, 1048beon-gil, Joongang-daero, Yeonje-gu, Busan, 47598 Republic of Korea; 3 Department of Environmental Administration, Catholic University of Pusan, 57 Oryundae-ro, Geumjeong-gu, Busan, 46252 Republic of Korea; 4 Coastal Disaster Prevention Research Center, Korea Institute of Ocean Science & Technology, 385, Haeyang-ro, Yeongdo-gu, Busan, 49111 Republic of Korea; 5 Department of Environmental Engineering, Inje University, 197, Inje-ro, Gimhae, 50834 Republic of Korea

**Keywords:** Pollution remediation, Hydrology, Mineralogy

## Abstract

This study evaluated the efficiency of the removal of heavy metals from contaminated water via adsorption isotherm and kinetic experiments on two composite mineral adsorbents, CMA1 and CMA2. The developed CMA1 (zeolite with clinoptilolite of over 20 weight percent and feldspar of ~10 percent, with Portland cement) and CMA2 (zeolite with feldspar of over 15 weight percent and ~9 percent clinoptilolite, with Portland cement) were applied to remove Cu, Cd, and Pb ions. Based on the adsorption isotherm and kinetic experiments, the adsorbents CMA1 and CMA2 exhibited high removal efficiency for Cu, Cd, and Pb ions in solution compared to other adsorbents. In the adsorption kinetic experiment, CMA2 demonstrated better adsorption than CMA1 with the same initial concentration and reaction time, and Cu, Cd, and Pb ions almost reached equilibrium within 180 min for both CMA1 and CMA2. The results of the adsorption kinetic experiments with pseudo-first-order (PFO) and pseudo-second-order (PSO) models indicated that the PSO model was more suitable than the PFO model. Comparing the Langmuir and Freundlich adsorption isotherm models, the former showed a very slightly higher correlation coefficient (*r*^2^) than the latter, indicating that the two models can both be applied to heavy metal solutions on a spherical monolayer surface with a weak heterogeneity of the surface. Additionally, the adsorbents CMA1 and CMA2 demonstrated different removal abilities depending on which heavy metals were used.

## Introduction

Substantial research has been conducted in the past 20 years on which materials are best for adsorbing heavy metals (Table 1). Garcıa-Sánchez *et al*.^1^ evaluated the heavy-metal adsorption capacity of clay minerals (sepiolite, palygorskite, and bentonite) from different mineral deposits with a reduction in metal mobility and bioavailability for remediation of polluted soils in the Guadiamar Valley. Erdem *et al*.^2^ studied the adsorption behaviour of natural clinoptilolite with respect to Co, Cu, Zn, and Mn ions and found that the adsorption was dependent on charge density and hydrated ion diameter, showing great potential for natural clinoptilolite to remove cationic heavy metal species from industrial wastewater. Ok *et al*.^3^ studied a mixture of zeolite and Portland cement (ZeoAds) as a substitute for activated carbon and tested its efficiency for the removal of heavy metals from aqueous solutions for wastewater treatment. Park and Hwang^4^ used adsorption tests to evaluate feldspar porphyry as an adsorbent for heavy metals in natural water. Nguyen *et al*.^5^ determined the adsorption behaviours of Cd, Cu, Cr, Pb, and Zn individually and collectively on an Australian natural zeolite with an iron coating (ICZ). He *et al*.^6^ carried out isotherms and kinetics studies using a synthesized zeolite from fly ash to investigate the adsorption capacity of heavy metal ions (Pb, Cu, Cd, Ni, and Mn) in aqueous solutions. Taamneh and Sharadqah^7^ evaluated the use of natural Jordanian zeolite (NJ zeolite) as a practical adsorbent for removing Cd and Cu ions. Lee *et al*.^8^ evaluated the adsorption performance of valuable metal ions (Cu, Co, Mn, and Zn) using a synthesized zeolite (Z-C2) from fly ash.

**Table 1:** Adsorption results of heavy metals by various absorbent materials.

Adsorbent materials	Absorbent dose to heavy metal solution (g/L)	Time (h)	Temp. (°C)			Adsorbed heavy metals (Maximum adsorption capacity, mg/g)							Authors
				Cd	Co	Cu	Cr	Fe	Pb	Mn	Ni	Zn	
Clays (sepiolites, palygorskites, and bentonite from different mineral deposits)	5	2	22	8.3		6.9						5.7	Garcıa-Sánchez *et al*.^1^
Clinoptilolite	20	5.5	30	9	14.4					4.2		8.8	Erdem *et al*.^2^
Activated carbon	40	24	25	6.4		12.3			18.4			7.2	Ok *et al*.^3^
ZeoAds (mixture of zeolite and Portland cement)	40	24	25	10.9		23.3			27			12.9	Ok *et al*.^3^
ICZ (iron-coated zeolite)	1–25	24	25 ± 2	7.24		9.33	5.47		11.16			6.22	Nguyen *et al*.^5^
Zeolite synthesized from coal fly ash	10	3	30	52.12		56.06			65.75	30.89	34.4		He *et al*.^6^
NJ zeolite (natural Jordanian zeolite)	20	24	22	25.9		14.3							Taamneh and Sharadqah^s^
Z-C2 (synthesized zeolite from fly ash)	1	4	30		77.7	94.7				57.7		51.1	Lee *et al*.^8^

Various researchers^9–13^ have characterized the chemical, surface, and ion-exchange properties of clinoptilolite. Zamzow *et al*.^11^ studied the inorganic cation exchange capacity of clinoptilolite and the selectivity series as follows: Pb^2+^ > Cd^2+^ > Cs^+^ > Cu^2+^ > Co^2+^ > Cr^3+^ > Zn^2+^ > Ni^2+^ > Hg^2+^. Jama and Yücel^10^ recognized a very high preference of clinoptilolite for ammonium ions over sodium and calcium ions but not over potassium ions. Mier *et al*.^12^ identified the interactions of Pb^2+^, Cd^2+^, and Cr^3+^ competing for ion-exchange sites in natural clinoptilolite. Regarding the ion exchange of Pb^2+^, Cu^2+^, Fe^3+^, and Cr^3+^ on natural clinoptilolite, Inglezakis *et al*.^13^ found that equilibrium is favourable for Pb^2+^, unfavourable for Cu^2+^, and of sigmoidal shape for Cr^3+^ and Fe^3+^. Since commercial clinoptilolite is relatively costly, mixtures of zeolite and other less expensive organic and inorganic materials, such as cement, clays, and polymers, have been formulated for specific pollutants^14^.

Recently, functionalized adsorbents such as nanocomposite materials have been prepared for harmful heavy metal ions and organic compound adsorption and have been used for diverse applications^15–23^. Moreover, natural mineral-modified absorbents have the advantage that they can be applied not only to the aqueous phase but also to the soil phase^24–29^. This study aimed to reveal the efficiency of the removal of heavy metals by applying modified natural minerals as adsorbents to contaminated groundwater. For this purpose, we evaluated the cation adsorption performance and adsorption equilibrium characteristics of the composite mineral adsorbents CMA1 (zeolite with clinoptilolite of over 20 weight percent and feldspar of ~10 percent, with Portland cement) and CMA2 (zeolite with feldspar of over 15 weight percent and ~9 percent of clinoptilolite, with Portland cement) through adsorption isotherms and kinetics. Specifically, we looked at how CMA1 and CMA2 performed in the removal of Cu, Cd, and Pb ions from polluted water.

## Materials and Methods

### Preparation and characterization of the adsorbents

**Figure 1:**
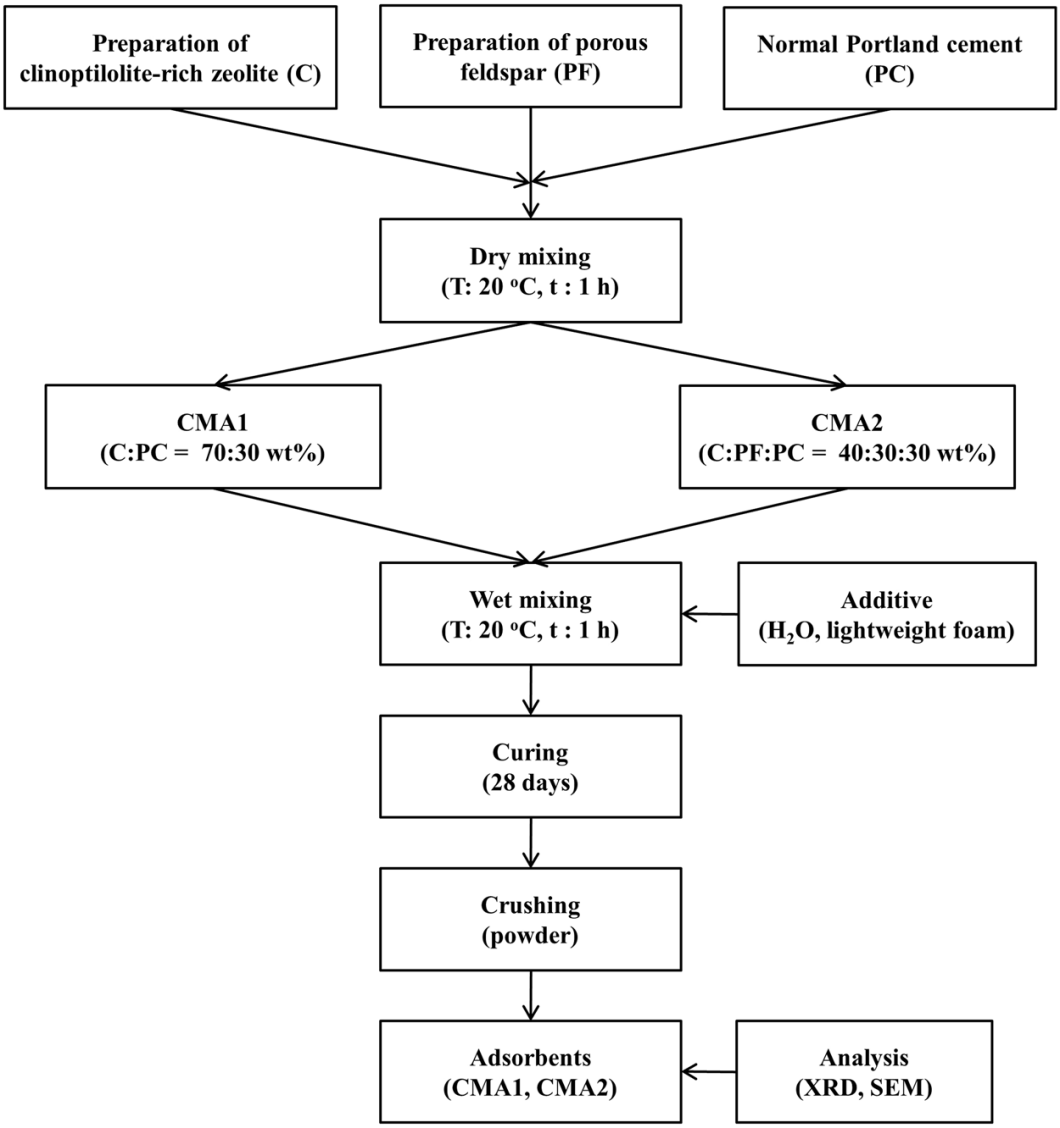
Procedure and analysis performed during preparation of the adsorbents CMA1 and CMA2.

The preparation procedure of the adsorbents CMA1 and CMA2 is illustrated in Fig. 1. All experiments were conducted in a 10-L polyethylene reactor equipped with a stirrer. To prepare the adsorbents CMA1 and CMA2, clinoptilolite-rich zeolite, slightly weathered feldspars showing a porous structure under a microscope, and normal Portland cement were prepared. Porous feldspar was prepared from weathered feldspar porphyry that was pulverized into 44-µm particles after being heated at 480 °C for 20 min. For the CMA1 adsorbent, a mixture of clinoptilolite-rich zeolite (C) and Portland cement (PC) at a ratio of C:PC = 70:30 wt% was cured for 28 days after adding water and lightweight foam. Finally, the CMA1 adsorbent powder was made by crushing the cured specimen. For the CMA2 adsorbent, a mixture of clinoptilolite-rich zeolite (C), porous feldspar (PF), and Portland cement (PC) at a ratio of C:PF:PC = 40:30:30 wt% was cured for 28 days after adding water and lightweight foam. Eventually, the CMA2 adsorbent powder was produced by crushing the cured specimen.

Crystallization and chemical characterization were performed by X-ray diffraction (XRD, Philips X’Pert-MPD System). XRD patterns of the samples were scanned on a powder diffractometer with Cu Kα radiation (λ = 1.54 Å) at a diffraction angle of 2θ in the range of 5–50° in 0.02° steps (3 s per step). The crystal morphologies of the samples were analysed by using scanning electron microscopy (SEM, Hitachi S-4200), with an accelerating voltage of 15 kV and a magnification of 20,000 times. The samples were coated with a thin layer of platinum and mounted on a copper slab using double-sided tape for the SEM analysis.

### Methodology

Batch tests were performed for adsorption isotherm and adsorption kinetic experiments using the two adsorbents (CMA1 and CMA2) and standard heavy metal (Cu, Cd, and Pb ions) solutions for the different ion adsorption performance and adsorption equilibrium characteristics. The adsorption isotherm and kinetic experiments were conducted to determine the adsorption characteristics and adsorption rate, respectively, of the heavy metal ions. Fifty millilitres of Cu, Cd, and Pb ion solutions and 0.05 g of CMA1 and CMA2 were placed in a 50-mL conical centrifuge tube (Falcon, 352070) and stirred at 200 rpm using a horizontal shaker (Vision, VS-8480S). For the adsorption kinetic experiment, 0.05 g of the adsorbent was added and stirred with 50 mL of a 1,000 mg/L heavy metal solution, and the residual concentration was analysed at reaction times of 30, 60, 90, 120, 180, 360, and 480 min. For the adsorption isotherm experiment, 0.05 g of the adsorbent was added to 50 mL of 50–1,000 mg/L heavy metal solution with stirring, and the residual concentration was analysed after 24 h of reaction time. The pH change experiments were conducted with 1,000 mg/L Cu, Cd, and Pb ion solutions at 25 °C. The initial pH in the solutions was adjusted to 3.0, 5.0, or 7.0 by adding 0.5 M HNO_3_ or 0.5 M NaOH solution. The pH values were measured using a pH meter (Istek AJ-7724, Korea).

Samples were taken at regular intervals and centrifuged (Vision Scientific VS-5000i2, Korea) for 3 min at 3,000 rpm. After centrifugation, the supernatant was filtered, and the Cu, Cd, and Pb ion concentrations were analysed by using an atomic absorption spectrophotometer (Perkin Elmer AAnalyst 100, Germany).

## Theory

### Adsorption kinetics

The pseudo-first-order (PFO) rate equation for the adsorption kinetics of solutes from a liquid solution that was proposed by Lagergren^30^ is:

1 $$\frac{dq}{dt}={k}_{1}({q}_{e}-q)$$

where *q* and *q*_*e*_ are the amounts of solute adsorbed (mg) per adsorbent (g) at any time and at equilibrium, respectively, and *k*_1_ is the PFO rate constant of adsorption. Integrating Eq. (1) for the boundary conditions *t* = 0 to *t* and *q* = 0 to *q* gives:

2 $${\rm{l}}{\rm{n}}\,\frac{({q}_{e}-q)}{{q}_{e}}=-{k}_{1}t$$

The pseudo-second-order (PSO) rate equation for the adsorption kinetics of solutes from a liquid solution proposed by Ho and McKay^31^ is:

3 $$\frac{dq}{dt}={k}_{2}{({q}_{e}-q)}^{2}$$

The integration of Eq. (3) for the boundary conditions *t* = 0 to *t* and *q* = 0 to *q* gives:

4 $$\frac{1}{{q}_{e}-q}=\frac{1}{{q}_{e}}+{k}_{2}t$$

where *k*_2_ is the PSO rate constant of adsorption. A linear equation can then be obtained from Eq. (4):

5 $$\frac{t}{q}=\frac{1}{{k}_{2}{q}_{e}^{2}}+\frac{1}{{q}_{e}}t$$

The plot of *t/q* versus *t* gives a straight line with a slope of 1*/q*_*e*_ and an intercept of 1*/k*_2_*q*_*e*_^2^, and then *q*_*e*_ and *k*_2_ can be evaluated from the slope and intercept, respectively.

### Adsorption isotherms

According to the formula by Vanderborght and Van Griekenm^32^, *Q*, the solute adsorbed (mg) per adsorbent (g), is

6 $$Q=\frac{V({C}_{i}-{C}_{e})}{W}$$

where *V* is the volume of the adsorbate (L), *C*_*i*_ is the initial concentration of adsorbate (mg/L), *C*_*e*_ is the concentration of the adsorbate after adsorption (mg/L), and *W* is the weight of the adsorbent (g). The experimentally derived isotherm can be fitted into the Langmuir^33^ and Freundlich^34^ adsorption isotherms, which represent, respectively, uniform adsorption energy onto the surface with no transmigration of adsorbate in the plane of the surface and the adsorption on a heterogeneous surface. The Langmuir adsorption isotherm is valid for monolayer adsorption onto a surface that contains a finite number of identical sites. The removal efficiency expressed as the percent of sorption is:

7 $${\rm{ \% }}{\rm{S}}{\rm{o}}{\rm{r}}{\rm{p}}{\rm{t}}{\rm{i}}{\rm{o}}{\rm{n}}=\frac{{C}_{i}-{C}_{e}}{{C}_{i}}\times 100$$

According to Langmuir, the amount adsorbed, *Q*_*e*_ (mg/g), is defined as:

8 $${Q}_{e}=\frac{{Q}_{m}{K}_{L}{C}_{e}}{1+{K}_{L}{C}_{e}}$$

where *C*_*e*_ is the equilibrium concentration of the adsorbate (mg/L), *Q*_*m*_ is the Langmuir constant related to the maximum monolayer adsorption capacity (mg/g), and *K*_*L*_ is the Langmuir isotherm coefficient related to the affinity of the sorbate for the binding sites. Equation (8) can be re-arranged into a linear form:

9 $$\frac{1}{{Q}_{e}}=\frac{1}{{Q}_{m}}+\frac{1}{{Q}_{m}{K}_{L}{C}_{e}}$$

Using the Freundlich equation, the amount absorbed, *Q*_*e*_ (mg/g), is:

10 $${Q}_{e}={K}_{F}{C}_{e}^{\frac{1}{n}}$$

Thus, linearizing Eq. (10),

11 $${\rm{l}}{\rm{n}}\,{Q}_{e}=\,{\rm{l}}{\rm{n}}\,{K}_{F}+\frac{1}{n}\,{\rm{l}}{\rm{n}}\,{C}_{e}$$

where *K*_*F*_ is the Freundlich isotherm coefficient or an approximate indicator of the adsorption capacity (mg/g), 1*/n* is a function of the strength of the adsorption in the adsorption process, and *n* is the adsorption intensity^35^. A value of *n* = 1 indicates that the partition between the two phases is independent of the concentration, *n* > 1 indicates normal adsorption, and *n* < 1 indicates cooperative adsorption^36^. A value of 1 < *n* < 10 demonstrates a favourable sorption process^37^. The constant change of *K*_*F*_ and *n* with an increase in temperature reflects the empirical observation that the quantity adsorbed rises more slowly, such that higher pressures are required to saturate the surface. For the determination of *K*_*F*_ and *n* by data fitting, linear regression is generally used to determine the parameters of the kinetic and isotherm models^38^. The linear least-squares method and linearly transformed equations have been widely applied to correlate sorption data, where the smaller the 1/*n* (or heterogeneity parameter), the greater the expected heterogeneity.

## Results

### Characterization of the adsorbents

**Figure 2:**
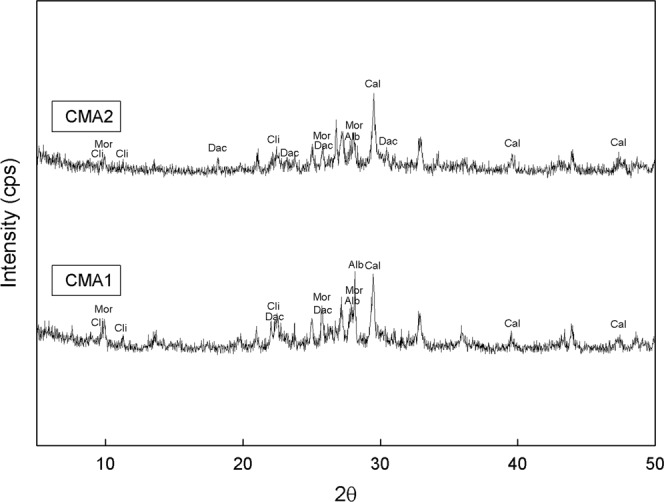
XRD peaks of the composite mineral adsorbents CMA1 and CMA2. Albite (Alb), Calcite (Cal), Dachiardite (Dac), Clinoptiloite (Cli), and Mordenite (Mor) have been identified.

**Figure 3:**
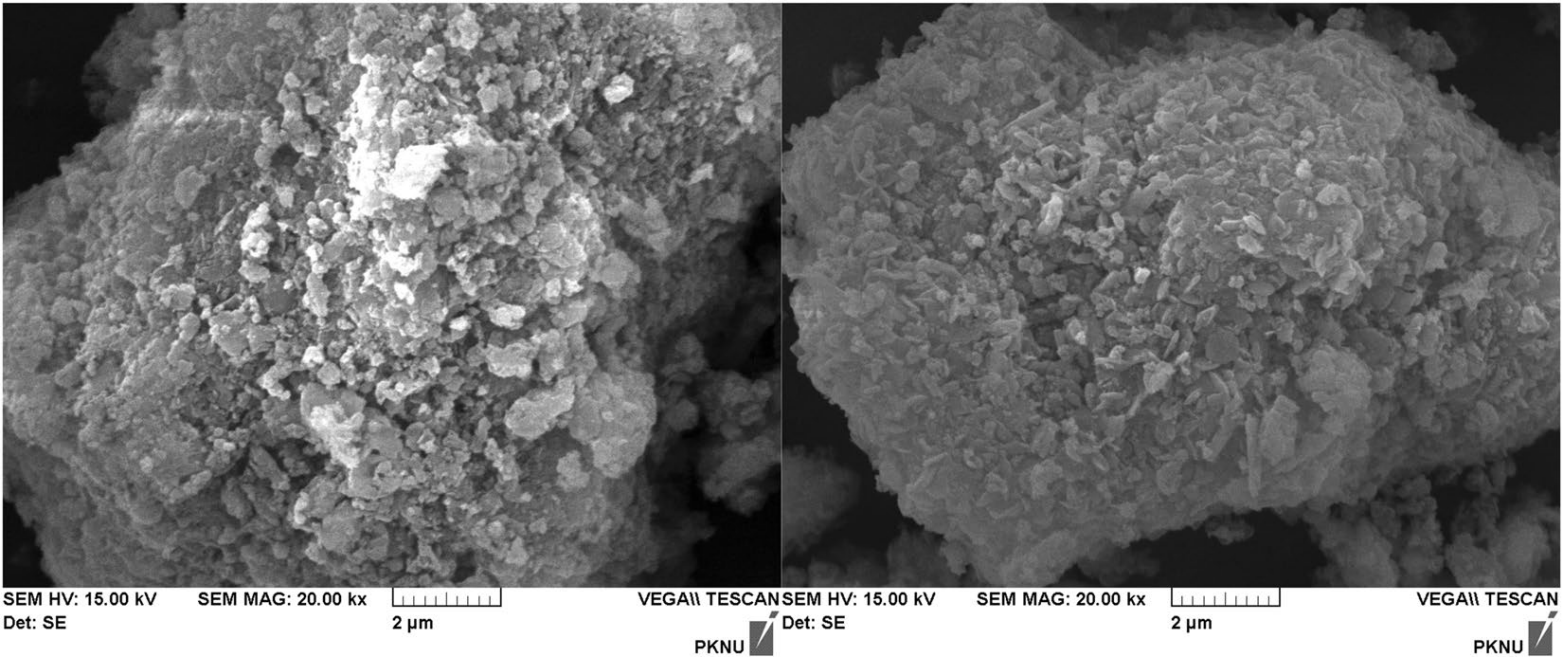
SEM images of the composite mineral adsorbents CMA1 (left) and CMA2 (right).

The composite mineral adsorbents, CMA1 and CMA2, are composed of clinoptilolite, feldspars, and Portland cement. XRD analysis showed that CMA1 and CMA2 consisted of albite, calcite, dachiardite, clinoptiloite, and mordenite (Fig. 2). Feldspar is reported to have a heavy metal adsorption capacity^39–41^. Clinoptilolite, with the chemical formula of (Na,K)_64_Al_6_Si_30_O_72_·nH_2_O, one of most abundant natural zeolites, is found in sedimentary rocks of volcanic origin and occurs with silicate minerals such as feldspar, quartz, other zeolites (members of the tectosilicates subclass), clays (members of the phyllosilicates subclass), and volcanic glass^42^. Clinoptilolite, along with heulandite and mordenite, has a high cation capacity^43^. Its tabular morphology shows an open reticular formation with easy access, formed by open channels of 8- to 10-membered rings. Feldspars, anhydrous aluminosilicates composed of potassium, sodium, and calcium, are structured by silicon and aluminium occupying the centres of the tetrahedrals of SiO_4_ and AlO_4_. These tetrahedrals are linked to other tetrahedrals at each corner, forming a 3-D, negatively charged framework. Potassium, sodium, and calcium within the voids of the structure can be exchanged with other cations. Portland cement is a very common solidification and stabilization material and is used as a supplement to clinoptilolite for adsorption purposes^44^. The SEM images of CMA1 and CMA2 presented amorphous porous particles, which are consistent with the aggregated forms of albite, calcite, dachiardite, clinoptiloite, and mordenite (Fig. 3). Overall, dachiardite, clinoptiloite, and mordenite can be considered major minerals involved in adsorption for heavy metal control^45–48^.

### Effect of initial pH on Cu, Cd, and Pb ions

**Figure 4:**
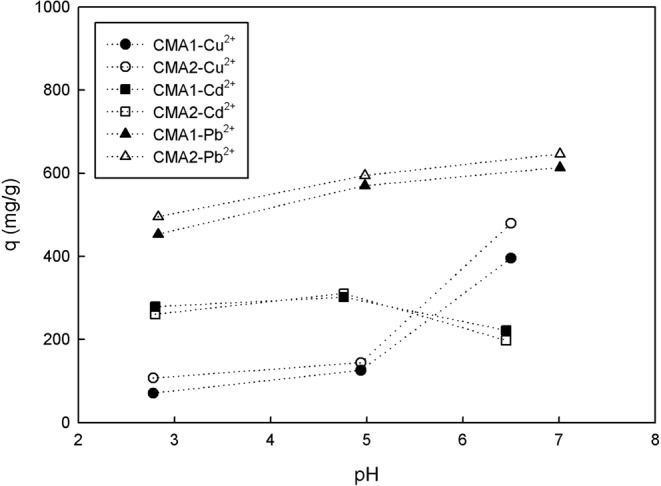
Effect of initial pH on the adsorption capacities of Cu, Cd, and Pb ions.

The adsorption of heavy metals is significantly influenced by the initial pH of the solution since the initial pH determines the surface charge of the adsorbent and the degree of speciation and ionization of the adsorbate^49–53^. The effects of the initial pH value were evaluated by the Cu, Cd, and Pb adsorption capacities in the solutions (Fig. 4). The adsorption capacities of Cu in the solutions were determined to be 71–107.5, 126–144.5, and 395.5–479.5 mg/g at initial pH values of 2.78, 4.94, and 6.50, respectively. The adsorption capacities of Cd in the solutions were 261–279, 302–311, and 198–222 mg/g at initial pH values of 2.80, 4.76, and 6.45, respectively. Additionally, the adsorption capacities of Pb in the solutions were determined to be 453.1–495.4, 570.2–594.6, and 613.8–646.5 mg/g at initial pH values of 2.83, 4.98, and 7.01, respectively. At pH < 5.0, Cu^2+^, Cd^2+^, and Pb^2+^ are the primary species in the Cu, Cd, and Pb solutions, respectively; the species vary with solution pH, and the adsorption of Cu, Cd, and Pb ions mainly involves divalent metal ions^54–56^. Based on the effect of the initial pH on Cu, Cd, and Pb ions, each of adsorption kinetic experiments was conducted at a pH value less than 5.0.

### Adsorption kinetic experiments

**Figure 5:**
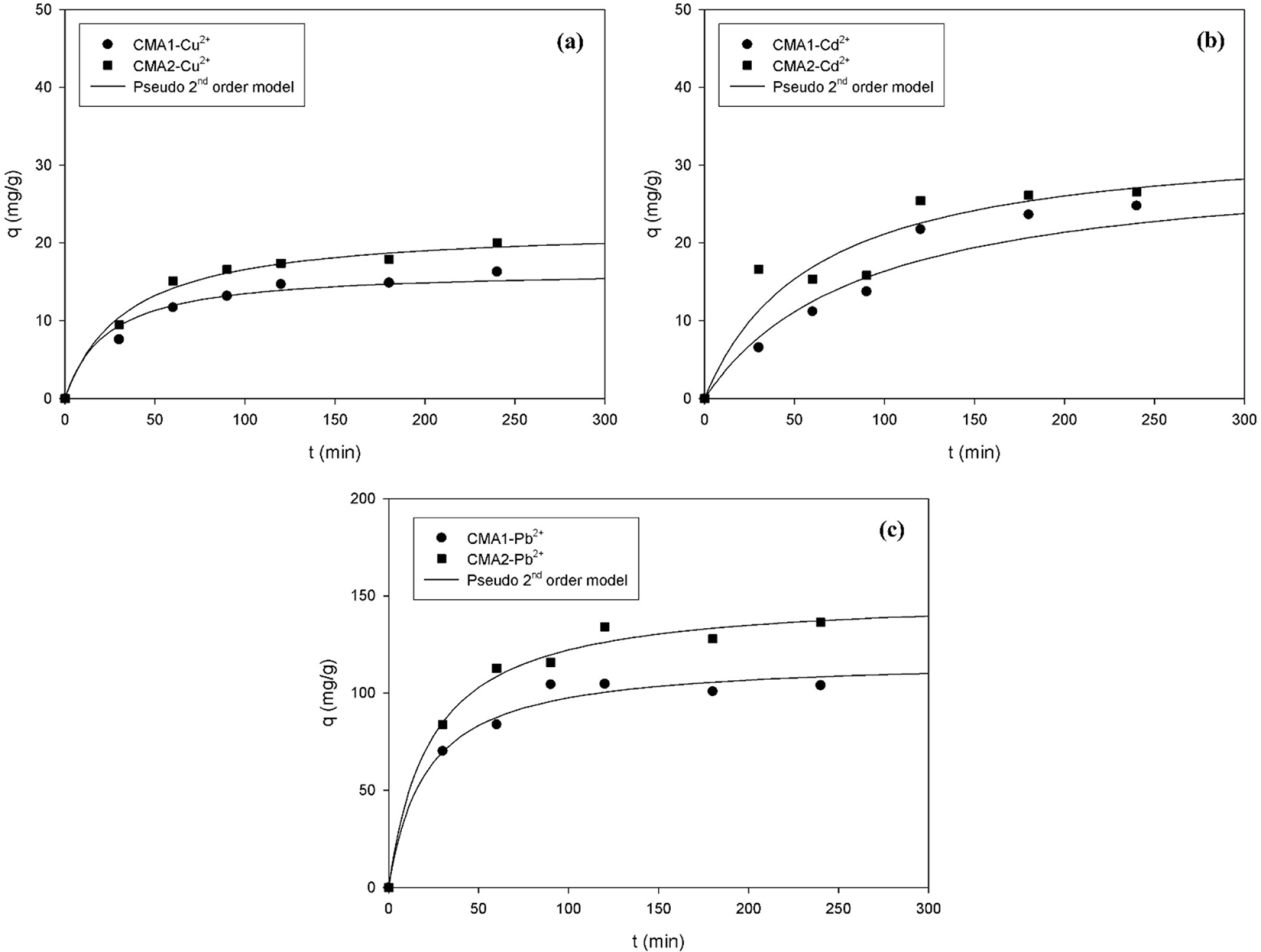
Adsorption kinetics for (**a**) Cu, (**b**) Cd, and (**c**) Pb ions on the adsorbents CMA1 and CMA2.

To determine the equilibrium reaction time, the adsorption of Cu, Cd, and Pb ions on the adsorbents CMA1 and CMA2 were plotted as a function of reaction time in Fig. 5. At the same initial concentration and reaction time, the CMA2 demonstrated better adsorption than the CMA1 (Table 2). The Cu, Cd, and Pb ions adsorption on the CMA1 and CMA2 had almost reached equilibrium within 180 min.

**Table 2:** PFO and PSO constants (*k*_1_, *k*_2_, and calculated *q*_*e*_*, q*_*e cal*_) for the adsorption of Cu, Cd, and Pb ions onto the adsorbents CMA1 and CMA2.

Ions	Adsorbent	*C*_0_ (mgL^−1^)	PFO			PSO		
			*k*_1_ (min^−1^)	*q*_*e cal*_ (mgg^-1^)	*r* ^2^	*k*_2_ (gmg^−1^·min^−1^)	*q*_*e cal*_ (mgg^−1^)	*r* ^2^
Cu	CMA1	1000	0.0308	18.2653	0.9515	0.00259	16.6001	0.9900
	CMA2		0.0165	18.4156	0.9040	0.00133	22.2067	0.9971
Cd	CMA1	1000	0.0231	32.5880	0.7690	0.00037	30.7407	0.9255
	CMA2		0.0100	22.6622	0.8529	0.00049	33.8665	0.9604
Pb	CMA1	1000	0.0221	98.4427	0.9416	0.00041	117.6717	0.9950
	CMA2		0.0117	81.0433	0.8143	0.00029	150.4259	0.9977

PFO and PSO models were applied to the results of the adsorption kinetic experiments for Cu, Cd, and Pb ions on the adsorbents CMA1 and CMA2. The PFO and PSO models demonstrated different determination coefficient (*r*^2^) values, between 0.7690–0.9515 and 0.9255–0.9977, respectively, indicating that the latter was more suitable than the former (Fig. 5). The adsorption capacities obtained from the experiments did not agree with the values estimated to reproduce the Cu, Cd, and Pb ions adsorption kinetics with the adsorbents CMA1 and CMA2 from the PFO model. However, the experimental results were similar to the values calculated from the PSO model, and the *r*^2^ values were also very close to unity. Therefore, Cu, Cd, and Pb ions adsorption by the adsorbents CMA1 and CMA2 could be more accurately explained by the PSO model than by the PFO model. Additionally, the adsorbent CMA2 was more effective in removing the heavy metals ions of Pb, Cd, and Cu than the CMA1, in decreasing order of their *q*_*e cal*_ values.

### Adsorption isotherm experiments

**Figure 6:**
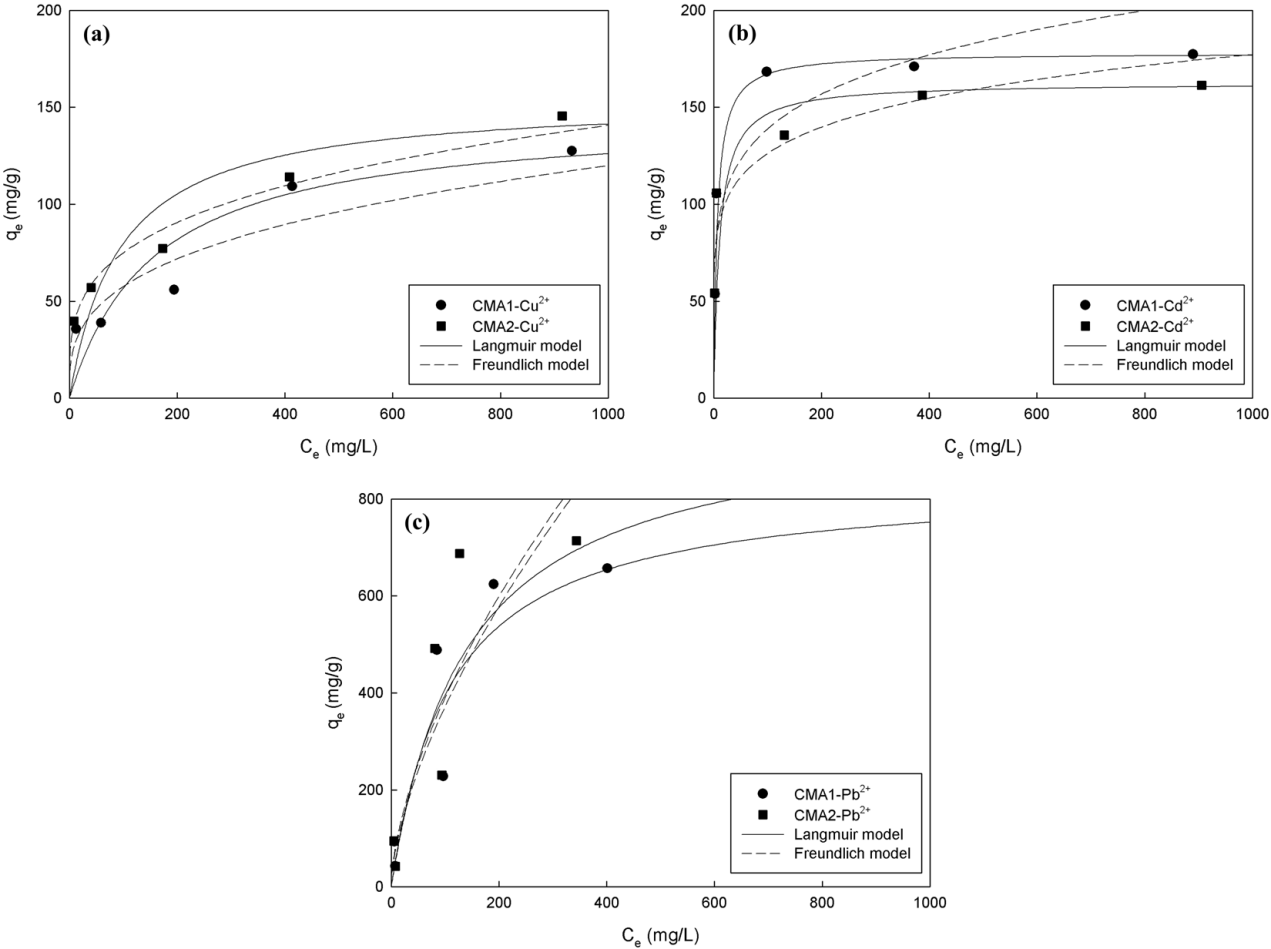
Adsorption isotherms for (**a**) Cu, (**b**) Cd, and (**c**) Pb ions on the adsorbents CMA1 and CMA2.

The adsorption isotherms for Cu, Cd, and Pb ions on the adsorbents CMA1 and CMA2 are plotted in Fig. 6. The isotherm parameters, *Q*_*e*_, *K*_*L*_, *n*, *K*_*F*_, and *r*^2^ obtained from the Langmuir and Freundlich isotherms are given in Table 3. The results of the adsorption isotherm experiment for Cu, Cd, and Pb ions on the adsorbents CMA1 and CMA2 were interpreted by using the Langmuir and Freundlich models. Table 3 and Fig. 6 show that the Langmuir model gave a slightly higher correlation coefficient (*r*^2^) than the Freundlich model, indicating that the two models could both be applied to the heavy metal solutions on a spherical monolayer surface with a weak heterogeneity of the surface. The adsorption capacities of Cu, Cd, and Pb ions by the Langmuir model were 145.84–154.71 mg/g, 162.58–177.99 mg/g, and 802.23–932.08 mg/g, respectively, while the adsorption capacities of Cu and Cd ions by Erdem *et al*.^2^ and Ok *et al*.^3^ were 9.0–23.3 mg/g using clinoptilolite (natural zeolite) and ZeoAds (a mixture of zeolite and Portland cement).

**Table 3:** Calculated maximum adsorption capacity (*Q*_*m*_) values for Cu, Cd, and Pb ions and parameters of Langmuir and Freundlich models using the adsorbents CMA1 and CMA2.

Ions	Adsorbents	Langmuir			Freundlich		
		*Q*_*m*_, mg/g	*K*_*L*_, L/mg	*r* ^2^	*K*_*F*_, (mg/g)(L/mg)^1/n^	*n*	*r* ^2^
Cu	CMA1	145.84	0.0064	0.9346	13.3202	3.1426	0.8617
	CMA2	154.71	0.0107	0.9722	21.3117	3.6593	0.9791
Cd	CMA1	177.99	0.1542	0.9998	61.9791	5.7072	0.8094
	CMA2	162.58	0.0921	0.9993	63.9752	6.7771	0.8261
Pb	CMA1	802.23	0.0132	0.9617	22.7868	1.6284	0.8948
	CMA2	932.08	0.0113	0.8917	22.2813	1.5406	0.8688

The maximum adsorption capacities (*Q*_*m*_) of Cu, Cd, and Pb ions by using the Langmuir model were determined to be 802.23 mg/g (CMA1) and 932.08 mg/g (CMA2) for Pb, 177.99 mg/g (CMA1) and 162.58 mg/g (CMA2) for Cd, and 145.84 mg/g (CMA1) and 154.71 mg/g (CMA2) for Cu, after dosing the adsorbents with only 1 g/L of the heavy metal solution; and hence, these values are significantly higher than those of the existing adsorbents as presented in Table 1. By comparison, an absorption experiment using a highly porous composite material with immobilization of an organic ligand onto silica monoliths^57^ that was performed to efficiently remove Pb ions in wastewater resulted in a *Q*_*m*_ of 204.34 mg/g, and an absorption experiment using a mesoporous composite material synthesized by the immobilization of an organic ligand onto mesoporous silica for effectively removing Cu ions in aqueous solution obtained a *Q*_*m*_ of 197.15 mg/g^58^.

## Discussion and Conclusions

In this study, the adsorption performance and adsorption equilibrium characteristics of the heavy metal ions were evaluated through adsorption isotherm and adsorption kinetic experiments. We applied the composite adsorbents CMA1 (zeolite with clinoptilolite of over 20 weight percent and feldspar of ~10 percent, with Portland cement) and CMA2 (zeolite with feldspar of over 15 weight percent and ~9 percent clinoptilolite, with Portland cement) to heavy metal (Cu, Cd, and Pb ions) solutions.

The adsorption kinetic experiments results showed that the adsorption of the heavy metal ions almost reached within 180 min. PFO and PSO models for Cu, Cd, and Pb ions on the adsorbents CMA1 and CMA2 resulted in different *r*^2^ values of 0.7690–0.9515 and 0.9255–0.9977, respectively, indicating that the PSO model was more suitable than the PFO model. Furthermore, the adsorbent CMA2 was more effective in removing the heavy metal ions of Cu, Cd, and Pb than the CMA1, in decreasing order of their *q*_*e cal*_ values.

The adsorption isotherm experiments showed that both the Langmuir model and the Freundlich model were applicable to the heavy metal solutions because the adsorbents had spherical monolayer surfaces with a weak heterogeneity of the surface. The maximum adsorption capacities of Cu, Cd, and Pb ions from the Langmuir model resulted were 802.23 mg/g (CMA1) and 932.08 mg/g (CMA2) for Pb, 177.99 mg/g (CMA1) and 162.58 mg/g (CMA2) for Cd, and 145.84 mg/g (CMA1) and 154.71 mg/g (CMA2) for Cu, dosing the adsorbents at only 1 g/L of the heavy metal solution. These maximum adsorption capacities were significantly higher than the maximum adsorption capacities of Cu, Cd, and Pb ions using other adsorbents, as presented in Table 1.

Furthermore, the adsorbent CMA2, including cost-effective natural feldspar, displayed better adsorption capacity than the CMA1. The results of this study can be applied to effectively remove heavy metal ions in contaminated water and wastewater since both absorbents (CMA1 and CMA2) showed excellent removal efficiency of heavy metal ions in solution. Future research will be focused on revealing the mechanism of the different performances on the heavy metal (Cu, Cd, and Pb) ions by the adsorbents CMA1 and CMA2, which is probably related to the feldspar content.
